# Motivation and Barriers to Maintaining Lifestyle Changes in Patients with Type 2 Diabetes after an Intensive Lifestyle Intervention (The U-TURN Trial): A Longitudinal Qualitative Study

**DOI:** 10.3390/ijerph17207454

**Published:** 2020-10-13

**Authors:** Sabrina K. Schmidt, Liv Hemmestad, Christopher S. MacDonald, Henning Langberg, Laura S. Valentiner

**Affiliations:** 1 Department of Sport, Physical Education and Outdoor Studies, Faculty of Humanities, Sports and Educational Science, University of South-Eastern Norway, 3800 Bø in Telemark, Norway; liv.hemmestad@usn.no; 2 The Centre of Inflammation and Metabolism and the Centre for Physical Activity Research, Rigshospitalet, University of Copenhagen, 2100 Copenhagen, Denmark; csm@chrismacdonald.dk; 3 Department of Infectious Diseases, Rigshospitalet, Section of Social Medicine, CopenRehab, Faculty of Health, University of Copenhagen, 1017 Copenhagen K, Denmark; Henninglangberg@gmail.com (H.L.); lauravalentiner@gmail.com (L.S.V.); 4 Department of Midwifery, Physiotherapy, Occupational Therapy and Psychomotor Therapy, Faculty of Health, University College Copenhagen, 2200 Copenhagen N, Denmark

**Keywords:** type 2 diabetes, lifestyle intervention, motivation, qualitative research, adherence, health belief model, self-determination theory

## Abstract

The purpose of this study was to explore and identify factors that influence motivation for and barriers to adopting and maintaining lifestyle changes in patients with type 2 diabetes, following participation in an intensive multiple-lifestyle intervention. Participants were recruited from the U-TURN trial, a one-year, intensive lifestyle intervention for type 2 diabetes patients. This study was conducted over time; informants were interviewed twice after the trial ended with a six-month interval between interviews. The qualitative data from these individual interviews were analysed using systematic text condensation with an inductive approach. Five themes emerged: Social support and relatedness, Achievement of results, Support from healthcare professionals, Identification with and acceptance of the new lifestyle and Coping with ongoing challenges. These are all important for maintaining lifestyle changes and diabetes self-management. Changing one’s lifestyle can be a constant, difficult struggle. For sustainable progress after an intensive intervention, the changes must be adopted and endorsed by patients and co-opted into their social setting. Belonging to an exercise group, confidence in managing the lifestyle adjustments and handling of challenges through continual support and professional diabetes treatment are crucial in maintaining and adhering to the new lifestyle.

## 1. Introduction

Type 2 diabetes (T2D) poses a major health challenge all over the world. The International Diabetes Federation has reported that 463 million people worldwide had diabetes in 2019, of whom 90% to 95% were diagnosed with T2D [1,2]. Moreover, it is estimated that by 2045, 700 million will be diagnosed as diabetic [1]. Type 2 diabetes is not curable, but approaches exist to prevent [3,4,5], slow disease progression [4,6,7,8] and reverse its effects [9] through lifestyle modifications and intensive lifestyle changes. Since behaviours such as poor diet and sedentary routines are among the strongest behavioural risk factors and a key aspect in treatment [10], lifestyle alteration is seen as the main method to overcome the T2D epidemic [1]. Multifactorial interventions have produced the most positive results [11], rather than single interventions or knowledge-based programmes [12,13]. Two systematic reviews by Knight [13] and Norris [7] confirm that such initiatives seem to enhance positive lifestyle changes and have positive effects on glycaemic control. The U-TURN trial, a 12-month lifestyle intervention including high exercise volume and dietary counselling was successful in reducing glucose-lowering medications in 73.5% of patients in the intervention group [14]. At one-year follow-up, the study showed that remission was achieved and maintained in a group with short disease history (a small subgroup of 14 patients out of 64) [15]. In general, few patients sustain lifestyle modifications and a meta-analysis indicates that the positive outcomes achieved decline after a trial ends [2]. To avoid relapse of the disease, maintenance of behavioural changes is essential. Adherence to lifestyle interventions is a specific challenge for future programmes, as concluded by a three-year follow-up of the Finnish Diabetes Prevention Study [5], while the U-TURN trial concluded that “further studies on maintaining a lifestyle change are needed” [15]. It is clearly difficult to sustain long-term lifestyle changes [7,16,17].

The term adherence (rather than compliance) emphasises a more active role for patients and their families in following a prescribed or recommended treatment. It has been defined as “the extent to which a person’s behaviour—taking medication, following a diet and/or executing lifestyle changes, corresponds with agreed recommendations from a health care provider” [18]. Factors that influence continual adherence have been identified as highly significant and recommended for prioritisation in future studies [10]. To better understand this topic, it is important to observe it from the patients’ perspectives. Motivation and barriers are central to attempts to improve adherence in diabetes treatment; therefore, factors influencing motivation and barriers to maintain the changes appear to be fundamental. Studies on patient views of lifestyle interventions have mainly been based on quantitative data [19,20,21]. A qualitative approach would provide valuable information, not only from the patients’ medical results but, perhaps more importantly, on their actual experiences that influence adherence. In order to tailor adequate and sustainable lifestyle interventions to individuals, it is crucial to fully understand the motivational factors and barriers that they encounter when an intervention ends. The aim of this study is to identify motivational factors for and barriers to creating and maintaining lifestyle changes in patients with T2D that took part in the intensive lifestyle intervention, the U-TURN trial. The research question is: what are the experiences of T2D patients, particularly in terms of motivation and barriers, in adopting and maintaining lifestyle changes after an intensive lifestyle intervention? The findings would provide a more comprehensive picture of how patients experience adherence to new routines after participating in an intervention. This study can be helpful in planning future initiatives that enable patients to gain sustainable health benefits through long-term adherence to lifestyle changes and effective treatment.

## 2. Materials and Methods 

### 2.1. Participants and Study Design

This is a qualitative, in-depth interview study with T2D patients enrolled in the U-TURN trial. The design is longitudinal, as data were collected over time, 12 and 18 months from baseline.

### 2.2. The U-TURN Trial

The informants were recruited from the intervention group in the U-TURN trial, a parallel-arm, single-blind, randomised clinical equivalence trial where the primary endpoint was glycated haemoglobin (HbA1c) monitored for 12 months [22]. The patients were divided (2:1) into a control group that received standard diabetes care according to Danish clinical guidelines [23] and an experimental group that underwent the U-TURN lifestyle intervention. The lifestyle intervention consisted of two main components: (1) increased levels of structured exercise for 240 to 420 min per week performed in groups throughout the entire project period and gradually reduced from supervised to self-monitored sessions and (2) anti-diabetic diet and individual meal plans, where patients attended three individual and 12 group sessions with a clinical dietician, as well as cooking classes. Four additional components were applied: increased levels of basal physical activity (10,000 steps a day), sleep duration (seven to eight hours), self-monitoring of behaviour, and diabetes management education and networking. These were digitally monitored daily by the patients, and data were uploaded to the intervention coordination centre. Comprehensive details and description of the trial and lifestyle components are reported by Ried-Larsen, Christensen, Hansen, Johansen, Pedersen, Zacho, Hansen, Kofoed, Thomsen, Jensen, Nielsen, MacDonald, Langberg, Vaag, Pedersen and Karstoft [22]. After the 12-month trial, guidelines and recommendations were given to the experimental group on post-intervention lifestyle goals, presented in Table 1.

### 2.3. Recruitment and Sample for the Qualitative Study

All patients from the intervention group in the U-TURN trial (*n* = 34) were informed about the study and given the opportunity to express their interest to participate in two interviews. From the list of those interested (*n* = 27), we selected informants to ensure diversity and sufficient rich data to answer the research question. We used the following criteria (1) representation of both genders, (2) diversity in residential areas (rural/urban sites), (3) informants from different intervention exercise groups and, furthermore, (4) enough informants to provide data saturation. Our final selection consisted of six informants, the characteristics of whom are presented in Table 2. All the informants were interviewed at 12 and 18 months post-baseline, after the end of the U-TURN trial intervention.

### 2.4. Individual In-Depth Interviews: Data Generation

To explore the informants’ experiences of adopting and maintaining lifestyle changes, data were collected over time. Qualitative studies are appropriate for this type of study, since they address context and particularities, while longitudinal studies focus on the exploration and interpretation of change over time and progress in social settings [24]. The 12-month interviews were conducted shortly after the intervention ended to track the informants’ behavioural adjustments and motivation achieved through the trial. A second batch took place six months later (the 18-month interviews) to identify barriers and facilitators to maintaining lifestyle changes in the longer term. Interviews were conducted face to face in the informants’ homes or in a quiet room at the research office. To ensure participation over time, the location was decided by the informants themselves [25]. Interview questions and method were developed and discussed with co-author LSV. Questions were used to guide the interview into descriptions of interest to answer the research question. Themes from interview guide is presented in Table 3. All interviews were arranged and conducted by the first author (SKS) and attended by an observer (KCW) to ensure validity; moreover, the observer was a more skilled interviewer providing basis for critical reflections on interview technique. The observer was at liberty to ask exploratory or clarifying questions if needed at the end of each session. In between each interview, a small analysis was made by the author. In this analysis focus was on reflections upon the interview by listening to the audio file, listening to the participants experience and descriptions, searching for clues of interest that warranted more attention in subsequent interviews, echoing the explorative approach [26,27]. Therefore, the interview guide was continuously adjusted. A further reason for adjustment of the interview guide was to encompass themes expressed by the informants and validate topics from one informant to another. Because of this approach, the first author (SKS) started the analysis at the first interview and had very good overview of the content in the data reception and a good basis to reflect on when data saturation was reached, and enough rich data was collected to answer the research question [28]. Furthermore, the ambition was to be explorative to present vital examples from people’s life worlds, not to cover the full range of potential available phenomena [27]. That is why a limited number of participants provides sufficient data for analysis and why six informants who all provided rich description was considered enough. The duration of the interviews was 60–120 min. Follow-up questions were carefully prepared, field notes on the environment and the participants’ behaviour, actions and emotions were obtained after each session as part of the dataset by the first author (SKS) and the observer (KCW). 

### 2.5. Data Analysis

All the interviews were transcribed verbatim, giving 242 pages of transcripts. To analyse the two sets of interview rounds (12- and 18-month follow-up), Malterud’s systematic text condensation was used [26,27] with an inductive approach by the first author (SKS). This method is inspired by Giorgi’s phenomenological analysis [29], modified by Malterud [26] and based on grounded theory [30]. First the 12-month material was analysed and later the 18-month dataset. Systematic text condensation contains four steps: (1) *Total impression*—*from chaos to themes.* All the transcripts were read to obtain an overall impression while searching for preliminary themes associated with the research question. These themes were discussed with colleagues, not for consensus but for a wider analytic approach. (2) *Identifying and sorting units of meaning*—*from themes to codes*. The transcripts were systematically reviewed line by line to identify text fragments (units of meaning) that contained information related to the research question. The units were coded in relation to the themes identified. This was a flexible process, where new themes emerged while others were removed as the data were analysed. (3) *Condensation*—*from code to meaning*. At this point, the data were reduced to a decontextualised selection of meaningful units sorted into code groups. Each group represented an analytic unit; the contents of the analytic unit (of meaning) were sorted into subgroups that revealed conflicts and nuances in the information. Each unit of meaning was then reviewed and condensed into an artificial quotation, retaining—as far as possible—the original terminology used by the informants. A condensate was created for each subgroup and a central quotation identified. (4) *Synthesising*—*from condensates to descriptions and concepts*. The condensed content was synthesised into an analytic summary that described the phenomenon grounded in the empirical data. Each description was validated by comparing it to the context of the interview and the data it was based on (reconceptualisation), as well as by searching all the transcripts for contradictory information. Furthermore, the results from the inductive approach were classified under the headings of each code group, which provided expressive statements of the most significant interpretations based on the data. Several key findings were identified during both the 12- and 18-month analyses, which complemented each other and provided insight into the informants’ experiences and how motivational factors influenced their adherence during and after the intervention. 

The final part of the analysis was conducted using a descriptive cross-sectional analysis that established the significant motivating factors and barriers over time based on both previous analyses. Emerging new themes were explored and identified by comparing and contrasting the data, following the most important findings and processes of the informants. 

### 2.6. Ethics

Principles of good ethical research practice were followed. Informants received verbal and written information about the purpose of the study. Written informed and signed consent forms were obtained at the first interview, and the informants agreed to the interview being audio-recorded. Furthermore, informants were reminded of their voluntary involvement and the possibility of withdrawing at any time without repercussions. The names of the informants have been replaced with their gender and age to protect and maintain anonymity. Under Danish legislation, qualitative studies do not require approval from an ethics committee.

## 3. Results

The same informants participated in the two rounds of in-depth, semi-structured interviews, conducted in August 2016 and February 2017. Two informants were not available for the follow-up sessions (18-month interviews) due to private matters, work pressures and illness. One of them provided comprehensive information through an email questionnaire and was therefore included. All the informants had reduced their use of anti-diabetic medication by the end of the lifestyle intervention, and no changes were reported in the 18-month follow-up interview. It became clear, however, that not all informants adhered to the recommended lifestyle goals, (self-reported routines compared to the guidelines displayed in Table 1). Changes in adherence and use of medication are presented in Table 4.

An overview of the 12- and 18-month analysis and how they formed the main themes and results in this study is presented in Table 5.

### 3.1. Achievement of Results

The informants all stated that their main motivation for participating in the intervention and changing their habits was to reduce their daily medicine intake and live an overall healthier life. When they invested time and energy in behavioural adjustments, feedback through results was a prominent factor in sustaining their efforts, results such as reduction in medication, improved fitness and physical form and experienced improved well-being/vitality. All the informants achieved a higher fitness level during the intervention and reduced their medicine intake; five out of six became free of all anti-diabetic drugs. The latter change was described as the strongest incentive for continuing their new lifestyle. One male informant explained: “I was motivated by not having to take medicine and having a better and longer life” (Male, 74). The informants experienced a range of psychological and physiological effects as a result of their lifestyle changes; they described being stronger, fitter, more energetic and better able to cope with stress. Understanding, observing and experiencing the outcomes of the behavioural adjustments added motivation to sustain the new lifestyle. One informant described the following: “I never had any discomfort from it, and that is a disadvantage, you do not have time to think back upon where you felt really bad (…) I feel that it motivates me, that my fitness has improved, that walking on stairs is not a problem, that I feel good., I have more energy, I get my sleep, I am fresh when I wake up. Such completely ordinary things, it sounds a little strange, but I actually wish I had felt bad at some point” (Male, 50). In addition, experiencing success and feeling more invigorated made the informants believe in themselves and become dedicated to achieving the goal of staying medication-free. At the 18-month follow-up interviews, no changes in dosage were reported, and medication-free informants stated that not having to take pills was still their main reason for maintaining the lifestyle changes, related to a feeling of shame. One informant reported: “Basically, it’s because I see that what I’m doing is working. I’m embarrassed that I haven’t done it before” (Male, 41). Despite living with T2D, the informants did not feel ill; therefore, the pill had become a result and indicator of their success. One male informant described how motivation to continue behavioural changes depended on observing consequences and achievement, an experience of a direct relationship between actions and outcomes. The informants found it important to be able to monitor their own progress through either self-perceived well-being, electronic data, amount of medication use or feedback from health professionals. We exemplify this by the statements from one informant: “I wear the clock that counts my steps. Usually I do more than 10,000 steps per day. I pay very much attention to this (…) if it says 7000 steps in the evening (…) it reminds me that I moved too little, and it motivates me to do extra the next day and then do 15,000 steps” and “it matters to me to be made aware of my situation by health professionals (...) You kind of wake up and it prevents me from going in a wrong direction” (Male, 41). 

### 3.2. Coping with Ongoing Challenges

All the informants had found it challenging to adopt the U-TURN lifestyle. The dietary restrictions were considered especially difficult while also following the “time-consuming exercise recommendations”. The implementation, and the lack of flexibility during the trial conflicted with informants’ work schedules, preferred daily routines and personal lives: “The hardest part was following the diet programme, all the recommendations and six meals a day. It was hard fitting the meals into my daily routine—that pressure all the time. I couldn’t comply; it felt so different all the time” (Male, 72). However, the informants felt able to cope with these challenges through their commitment to the project, support from the health professionals, exercise group and their family, along with their personal motivation to stay medication-free. Challenges were experienced as never-ending six months post-intervention. They came in different forms, challenging the informants’ ability and motivation to adhere. Two of the informants struggled with injuries that made it difficult to continue the recommended exercise. One managed to maintain regular exercise sessions’ as she lived near a gym, where a personal trainer helped her adjust the programme and perform rehabilitation, keeping her motivated and enabling her to adhere to the lifestyle. Informants experienced various internal and external struggles, both in managing their diabetes and in their social lives: deaths in the family, conflicts between social norms and intervention guidelines, lack of support from the healthcare system, personal problems and work pressures. Several managed to cope with these difficulties, while others did not. The most adherent informants succeeded in maintaining their new lifestyle by acknowledging that the constant challenges in life itself would interfere with their ability to cope with diabetes: “There’s been a lot of pressure at work. I pull myself together and tell myself it’s going to get better, otherwise I might just find another job. I have a personal life that I need to take care of” (Female, 49). All the informants described how their lives in general had been influenced by the behavioural changes and how they dealt with this in their own ways. Lifestyle alterations and management of T2D is experienced as a constant struggle, where sustaining continuous energy and focus is important in coping with never-ending challenges and staying positive. A male informant describes how he struggled: “So much has happened, I lost a close friend, my shoulder is injured and I have not been able to exercise the last three months, someone broke into my house, my sister died (…) with all that, sometimes I feel like just giving up” (Male, 74).

### 3.3. Social Support and Relatedness

All the informants viewed their participation in the intervention as a unique chance to receive support in their processes of changing behaviour and living a healthier life. Joining the project represented a binding promise that would keep them committed and obligated to adhere. The exercise group and the coaches positively influenced the informants’ motivation to exercise regularly by making them feel committed and part of a social community. The fixed schedule and the knowledge that one is expected by others to attend and participate made the informants prioritise their exercise sessions throughout the intervention period. Furthermore, they believed that following a fixed schedule in the company of others made them exercise harder and stay more focused. With help from the coaches, they developed skill and confidence in exercising, which resulted in greater incentive to perform the exercise sessions. A male informant noted: “When it’s just me, it’s easier to just stay at home, on the sofa. Having commitments and an exercise group motivates me” (Male, 41). During the transition from a structured intervention to independent management of one’s lifestyle, disease self-care and exercise, the informants faced various difficulties in establishing new daily rhythms and routines. Six months after the trial ended, all of them reported a continual focus on maintaining increased daily physical activity, but the recommended exercise guidelines were more challenging. Lack of energy, personal problems, injuries and absence of a group to exercise with were typical barriers, with the last factor proving to be the most prominent. One female informant stated: “I miss it a lot, both the group itself and exercising together. When I’m all alone, I don’t make it out of the house (…) If someone didn’t show up during the intervention, we would call him, and we never started until everybody was there; it was amazing” (Female, 69). Several informants experienced the same loss of support and exercise group; they described the intervention as being in a bubble, which was later burst. Those who struggled the most to continue regular workouts had difficulty finding new exercise partners in their local community post-intervention, after having the other participants in a temporary structure. All the informants were motivated by participation in a group setting and having someone or something to relate and commit to. Finding and engaging with such drivers, however, proved to be a challenge, especially for the older informants who had had no previous exercise partners and little support from their family; they had been highly motivated by being held socially accountable by their exercise partners.

### 3.4. Support from Healthcare Professionals

The medical professionals who conducted the intervention provided the informants with support, knowledge and information that they had not previously received. All the informants felt safe and confident in the way that their diabetes was being treated and monitored. The nurse and coaches in particular played an important role, due to their regular interactions with the informants and their understanding of each individual’s abilities and needs. The specialised treatment and support were highlighted by the informants, who felt taken care of, regulated and well-informed, as the nurse could answer any questions they had. The professionalism of the trial and the personnel kept them committed through regular personal meetings and diabetes tests; it also provided steady results and motivated them to work consistently towards their goal of reducing medicine intake and staying well-regulated. One informant noted: “I’ve tried the best I could. When I asked my doctor, everything was forbidden. This year I found out what it’s all about” (Female, 49). After returning to primary care with their general practitioners, the informants described a lack of support and difficulty coping with the disease on their own. All of them were advised to continue regular check-ups with their doctors, but none had done this. More than half stated that their general practitioners did not handle their illness with the same seriousness as the professionals involved in the U-TURN trial. One informant reported that she still did not take any medication, but was unsure if her vital signs were normal, because her doctor declined to perform regular tests. Another medication-free informant was informed that his routine check-ups were a waste of time and resources, and that he could consult a doctor every second year. He noted: “I liked being checked; maybe it’s just me being selfish. It was really nice to have that security: you go there, get checked and then you’re more aware of things and reflect on them” (Male, 41). Not only did the tests offer the experience of proper treatment and management of the disease, they also motivated the informants to remain focused and maintain their lifestyle changes, in addition to feeling safe. In the interviews, the informants emphasised a need for acknowledgement through continuous tailored support and monitoring, even when they were coping with their illness according to specific or personal guidelines.

### 3.5. Identification and Acceptance of the New Lifestyle

Managing and understanding diabetes was a challenge to most of the informants. Instead of the trial-and-error approach they had previously used, they now described greater confidence in their capacity to self-manage at the end of the intervention. All of them exhibited a change in attitude and ways of prioritising. Three informants in particular were able to plan according to their goals and displayed signs of the new lifestyle being part of their lives and self-image: “I think it’s become a habit, and I know I’m doing something good for myself and I like it” (Female, 49). Although they all experienced benefits from both exercise and diet, few endorsed the new lifestyle as their own following the intervention. The three informants who were most successful in sustaining the changes expressed acceptance and identification with the lifestyle they had adopted, which were lacking in their “non-adherent” counterparts. The most adherent informants described determination to reach their goal and continue the new lifestyle, as well as heightened belief and confidence in themselves despite the challenges they had faced. Their behavioural adjustments had become an active choice, creating new routines that they enjoyed and could not live without: “I feel bad if I don’t do my exercises, not in a depressive way, but I made a commitment to myself. I think about it every day, and I always keep an eye on my watch that counts my steps. It’s just part of me now, like you have to breathe every day, and then you just have to do a little extra” (Male, 50). Those who had embraced the necessary modifications as part of their new routines, as opposed to viewing them as unattainable guidelines, exhibited greater adherence in sustaining change, but the new lifestyle was still described as highly demanding.

## 4. Discussion

Our qualitative exploration of the informants’ experiences over time following participation in the U-TURN lifestyle intervention has enabled us to identify and understand factors that influence motivation and barriers to adopting and maintaining lifestyle changes. Various theories and models have attempted to explain health-related behavioural change, with varied and limited success [31]. This suggests that evidence-based research does not support one single, exclusive explanation of this phenomenon [32,33,34]. With the complexity of lifestyle and behaviour change in mind, the results from this study are discussed here in relation to the health belief model (HBM), self-determination theory (SDT) and relevant research on the topic. By relating the informants’ experience to behaviour and motivation theories we gain more knowledge about behaviour in relation to health, motivation and habits, and obtain access to perspectives that the U-turn trial and quantitative research are limited in reaching. The HBM has been selected as a framework due to its specific focus on behaviour change and improvement of one’s own health. Being diagnosed with T2D a large part of the treatment relies upon the individual, thus the patient becomes central in their own treatment. The HBM recognises the complexity of health-related behaviour change and can be used as a framework to understand human behaviour and actions towards living a healthier life and factors influencing health-related behaviour change [35]. The HBM has been applied in various health research settings and in diabetes research specifically, it has been applied to research self-care, adherence and metabolic control and motivation [20,36,37]. Where the HBM helps understand what leads up to “likelihood of taking recommended health actions” [35], it offers limited attention to maintaining health-related behaviour change. SDT is specifically identified as a useful theory to address health behaviour maintenance [38]. A review of behaviour theories by Kwasnicka, Dombrowski, White and Sniehotta [38] presents five themes relevant to maintenance showing that SDT has its contribution to “maintenance and motives” [38] and therefore especially relevant to this study and its research question, in order to understand the informants experience maintaining their lifestyle change. 

### 4.1. Ready to Change

The HBM [39,40] provides a framework to explain the informants’ behavioural changes towards a healthier life with diabetes. It consists of six concepts, which in combination lead to behavioural change: (1) *susceptibility* and (2) *severity* (where (1) and (2) combined represent perceived threat), (3) *perceived benefits* (belief in the efficacy of the advised action in reducing risk or severity), (4) *perceived barriers* (tangible and psychological costs of an action), (5) *self-efficacy* (confidence in one’s ability to take action) and (6) *action cues* (triggers for decision processes that activate readiness) [41]. The participants all believed that their main motivation for participating in the intervention and modifying their lifestyle was to reduce their daily medicine intake and live overall healthier lives. They were aware of the need for change, accepted responsibility for self-management and recognised the importance of support in the process. The model states that individuals will take health-related actions if: (1) they believe that negative health effects can be avoided and (2) they have faith in their own abilities to take the relevant actions [41]. *Perceived susceptibility and severity* are reflected in the informants’ desire for better and healthier lives with T2D and avoid further progression and complications from the disease. According to the HBM, this perception itself is important for behavioural change motivation. The intervention project itself represents “*cues to action*” as an opportunity to initiate a lifestyle modification with support in the process. *Perceived barriers* are reflected in the challenges facing the informants in maintaining the new lifestyle, such as time and energy consumption, work and injuries. Finally, *perceived benefits* are manifested in their statements about continuing the behavioural changes; they are encouraged by the results and wish to maintain the outcomes achieved. Research indicates that patients with T2D are generally reluctant to adapt their lifestyle, often ignore the severity of the disease and that adherence to lifestyle treatment is low [42,43]. Informants in the present study acknowledged the seriousness of the impact of the disease, but highlighted the *cues* to initiate lifestyle changes: being giving “a once in a life time opportunity” and support in a process where they have a feeling of commitment and relatedness. The importance of feedback and recognition for following the guidelines for the desired lifestyle is evident. In this case, the most prominent outcome and motivator of change was independence from medication. Not having to take the pills figured as an important symbol of their efforts. The informants’ drive and ability to maintain behavioural adjustments were highly influenced by their experience of results and achievement; this indicated a direct relationship between their actions and the outcomes. Another motivator was the ability to monitor progress through e.g., the symbolic action of not taking any pills, feelings of well-being, checking electronic data on their efforts or receiving feedback from healthcare professionals. Being aware and pleased with the outcome has also showed significance in the research by Rothman and colleagues, where satisfaction with behaviour change has been identified as a key determinant for maintaining behaviour change [44].

Commitment and social support can be characterised as elements of extrinsic motivation according to SDT [45], and it is argued that an individual cannot change and maintain changes based exclusively on extrinsic motivation. After the intervention, a clear tendency emerged over time, where the informants who identified with and expressed delight in the new lifestyle managed to sustain the behavioural adjustments. By contrast, those who were “non-adherent” or experienced relapse struggled to live the life they desired and felt guilty for not living by the recommendations.

### 4.2. Identification and Intrinsic Motivation

This study has revealed that identification with and acceptance of a new lifestyle led to greater adherence. Overall, this is in line with the SDT. The SDT model distinguishes between intrinsic and extrinsic motivation, where the latter is defined as the performance of an activity due to its inherent satisfaction. With this type of incentive, an individual is more likely to maintain long-term behavioural changes [46,47]. According to SDT, relatedness, competence and autonomy are the three basic needs that must be satisfied to ensure optimal development, integrity, well-being and intrinsic motivation. None of the informants in this study were entirely intrinsically motivated, but their drive changed on the scale of motivational regulations of SDT (degree of internalisation) and can be categorised into more than one subtype. The most successful informants had undergone a change in motivation towards the intrinsic end of the spectrum (integrated regulated/identified regulation) and were driven by self-determined motivations. Diet and exercise had become a part of their daily routine and the behavioural aspect of their self-image; they expressed greater confidence in their abilities to manage the disease. By not simply accepting unfamiliar guidelines, but rather consciously aligning the intervention guidelines with features of their daily rhythms and habits, the adherent individuals managed to make the new lifestyle their own. Informants who embraced the necessary behavioural modifications as part of their new lifestyle displayed greater adherence in maintaining change. This suggests that the most successful informants had their basic psychological needs fulfilled. However, “non-adherent” informants were more extrinsically motivated; this worked and motivated them to change their lifestyle behaviour during the intervention when they were part of a community and felt obliged to adhere. However, following the intervention, they had difficulty in finding external factors that could motivate them and had little intrinsic motivation to take responsibility for their own “healthy life project”. Many described challenges in finding joy, relatedness and support in their process of maintaining the new lifestyle.

### 4.3. Social Support and Relatedness

The analysis indicates that, in order to maintain structured daily exercise following such an intervention, it is important to identify with and become engaged in activities and supportive communities to which one can relate. This is vital to sustaining motivation and, as reported by the informants, to improving the quality of exercise. A study from 2006 has revealed that individuals who experience positive relatedness when exercising were more autonomously motivated and more able to sustain exercise sessions over time [48]. This suggests that creating an enjoyable setting and fostering emotional support makes people more likely to attend exercise sessions, as they may look forward to interacting with friends or receiving praise from instructors [48]. This study has demonstrated that informants who returned to or joined activities and groups where they felt related were most successful in maintaining structured exercise sessions and overall adherence to an active lifestyle, in accordance with SDT [45]. Those who had had no previous exercise partners were challenged, which suggests an inadequacy of the time-limited intervention: relatedness was satisfied in a temporary and unsustainable manner. According to SDT, variations in the three basic needs affect the well-being and functional capacities of individuals [45]. Some informants described the trial as being in a bubble; they were suddenly left alone and missed their exercise group, because of a lack of relatedness. Maintenance of lifestyle changes was difficult when informants experienced changes in their satisfaction of the three basic needs. Their adherence to a new lifestyle became vulnerable when settings or relationships were lost or altered, thus affecting their relatedness. More specifically, when adopting the lifestyle changes on a long-term basis, it was difficult for the informants to find a group of like-minded people to provide the motivation to adhere to their desired lifestyle. It remains a challenge to determine how health promoting lifestyle interventions can bridge this gap, whereby the support, relatedness and autonomy participants receive in a programme can somehow continue in their lives after completion of the programme.

### 4.4. Healthcare Professionals and Autonomy Support

Support from healthcare professionals (diabetes nurse and trainers) in this study proved to be an important motivator for lifestyle change over time. This is confirmed by research on effective ways to increase physical activity in T2D patients, which emphasises either a consultative [49] or counselling approach [50] as the key to success [51]. During the intervention, the informants felt encouraged in their processes of behavioural adjustment and self-management. This is particularly important according to SDT, which describes it as autonomy support [45]. The most essential elements for making meaningful changes are being independently motivated and perceiving oneself to be competent in lifestyle change [45]. The informants expressed a need for continued support from health professionals after the end of the personalised assistance (regular testing, monitoring and feedback) provided by the project. In particular, the lack of support from their regular doctors influenced their motivation and well-being. Research has characterised living with T2D as an unremitting inner struggle [52] and has addressed the importance of coping with the illness [53]. This study has revealed that the management of these difficulties greatly influences individual abilities to maintain lifestyle changes, and that feeling overwhelmed and lacking the necessary energy are demotivating factors. Those who coped most successfully with the ongoing struggles actively sought fulfilment of their basic needs for autonomy (medical help from professionals and personalised counselling at a gym), relatedness (exercise group and family) and competence (goal setting and maintaining results), where the last of these describes the degree to which an individual feels capable of achieving his or her goals. According to the SDT model, the satisfaction of all three basic needs is facilitated by autonomy support [45]. Other studies have highlighted the importance of continuous personalised encouragement to strengthen motivation for self-management by performing and enhancing the required diabetes care [54]. Such encouragement is indispensable to patients with chronic illness [55]. This study has demonstrated that acknowledgement, consistent and tailored support and monitoring are vital, even for those managing their disease according to personal and healthcare guidelines. The motivation to sustain the desirable lifestyle is highly influenced by one’s coping ability and belief in the professionals and methods involved in the treatment.

In this study, a gap was uncovered between expectations for the importance of lifestyle change counselling and actual practice in primary care. One informant described himself as being selfish regarding consultations with his doctor, because he preferred regular appointments, which enhanced his motivation to adhere to a medication-free lifestyle. Many general practitioners do not focus on motivational factors and how these affect treatment and adherence, and one study identified primary care physicians’ lack of confidence in facilitating behavioural change in patients, and their lack of incentives to make the necessary time commitment [56]. The importance of a positive relationship between patients and healthcare providers has also been noted in the DAWN study, which suggests that having a nurse on the premises was positively correlated with adherence to both medication and lifestyle regimens [57]. The organised exercise sessions were described as the easiest change to implement during the intervention. Six months after the trial ended, however, this became the most challenging modification to sustain. Three informants had maintained structured exercise four to six times a week, though they viewed it as a struggle. The adherent female informant who experienced major challenges from injuries and work-related stress stated: “It’s really hard, and if other people say anything else, they’re lying”. This suggests a certain pressure on the informants to live up to the behavioural recommendations.

Depression has been more commonly observed in patients with diabetes than in the general population [58], and approximately 18% of T2D patients experience high levels of illness-related distress [59]. This refers to significant negative psychological reactions to the demands of diabetes management and encompasses a broader range of emotions than the mere presence of symptoms [60]. Living with this disease is challenging, and behavioural alterations affect not only the daily routines of T2D patients but also their families. These challenges influence individuals’ well-being and increase the risk of diabetes distress. Adherence to the U-TURN lifestyle is tough and requires dedication, but it is important to recognise that even small lifestyle adjustments can have a positive impact on patients’ health. Some individuals have said that it is unrealistic to perform structured exercise six days a week in addition to daily physical activity and an anti-diabetic diet, and it is no secret that even non-diabetic relatively active individuals would find this lifestyle challenging. This must be considered when facilitating major changes as part of diabetes management, in order to enable lifestyle recommendations to be followed in a sustainable and realistic approach that ensures the mental well-being of the patients.

This study has its limitations. The analysis was performed by one researcher (SKS). It would have strengthened the analysis with more than one researcher—not for consensus, but to create a wider analytic space [27]. To strengthen the analysis the process and preliminary findings was discussed with co-authors LSV and LH continuously during the whole process. As the informants were part of a clinical trial, the generalisability of the findings is restricted. Recruitment of newly diagnosed patients, as well as the geographical setting and intensity of the intervention, is difficult to reproduce in general practice. This study is based on the lived experience of a small number of patients, yet they all provided rich data about the phenomenon of changing and maintaining their lifestyle to better manage their disease. The study aim was not to extend the findings derived from selected individuals to the general population, but rather to enable them to be transformed and applied to similar situations in other contexts.

## 5. Conclusions

This research has suggested the difficulty of changing and maintaining lifestyle. Five motivating factors that influence such post-intervention adjustments were identified as: Achievement of results, Coping with ongoing challenges, Social support and relatedness, Support from health care professionals and Identification with and acceptance of lifestyle. To ensure sustainability, the behavioural modifications must be adopted by patients and endorsed by their family and friends. Relatedness to an exercise group, feeling competent and successful in handling the changes, and support from health professionals appear to be key factors in adhering to a new lifestyle. The last element is vital and comprises two main points: (1) autonomy support and (2) regular tests that provide visible feedback and build trust in the specialised treatment process.

Further research is recommended on how optimal behavioural adjustments can be facilitated in patients’ everyday lives and how intensive lifestyle changes can be implemented with consideration for diabetes distress and patient well-being.

### Practice Implications

The results of this study suggest a number of practical implications. First, the lack of support and regular treatment after the intervention needs to be addressed. The encouraging effect of consistent check-ups for diabetes patients and how these enable them to feel safe and adhere to recommended behaviours should not be underestimated. General practitioners and relevant medical professionals need to develop competence in motivational strategies [54] or participate in patients’ lifestyle interventions as their main healthcare provider. When a T2D patient manages, through intensive behavioural adjustments, to reverse the disease, this condition is not stable. Blood glucose levels may change with any variations in exercise and dietary habits. Patients with T2D should still be taken seriously, even with normoglycaemia and no medical reason to take diabetes medication. It is recommended that for partial or complete remission of the disease for fewer than five years, patients should be screened for diabetes-related complications at the same frequency as when the illness was present [9]. Individuals who manage their T2D through physical activity and structured exercise may be highly dependent on their physiological condition; their motivation and adherence may thus become fragile in the case of injury. This echoes the need for autonomy support that enables patients to manage the ongoing challenges of lifelong treatment. An intervention is a start, a point of departure, but more focus should be directed at the time after the intervention, at how healthcare providers can help and support the patients to make the positive changes long-term and effective.

## Figures and Tables

**Table 1:** Lifestyle guidelines recommended to experimental group after the U-TURN intervention.

		Frequency and Duration	Intensity
**Exercise**	Cardio training	4–6 times a week, 30–60 min	Average 70–90% of max pulse. Variation between continually exercise and interval exercise
	Resistance training	2–3 times a week, 30–45 min	Variation in sets and repetitions, 3–5 set and 6–12 repetitions
**Daily physical activity**	Daily physical activity can be walking, cycling, playing with kids or grandkids, house or garden work. Try to do 10,000 steps a day as a minimum.		
**Diet**	(1) Avoid sweet food (especially refined sugar) and artificial sweeteners, (2) minimize the intake of processed food and 3) eat as a minimum 400 g vegetable pr. day.		
**Sleep**	Sleep needs to be prioritized in both quality and quantity. Try to get 7–8 h of sleep pr. night		

**Table 2:** Characteristics of informants.

Informant	Gender	Age	Disease History (Years)
Informant 1	Female	49	7
Informant 2	Female	56	2.5
Informant 3	Female	69	8
Informant 4	Male	50	4
Informant 5	Male	41	4
Informant 6	Male	74	6

**Table 3:** Themes from interview guides.

12 Month Interview Themes	18 Month Interview Themes
Informants description of themselves	Status since the intervention stopped
Experience in being in a trial	Adherence to lifestyle changes
Motivation to change lifestyle	Challenges in adherence
Previous attempts to change lifestyle	Sustained change or relapse
Exercise history and experience related to exercise in the trail	Individual specific follow-up questions
Motivation and barriers to adapt the U-TURN lifestyle (exercise, daily physical activity, diet, sleep)	Experience in maintaining recommendations in exercise, daily physical activity, diet and sleep
Use of anti-diabetic medicine	Reflections about adherence
Support and level of importance	Proposal on how to increase adherence to lifestyle in lifestyle interventions

**Table 4:** Self-reported use of antidiabetic medication and level of adherence over time.

	Reported Use of Anti-Diabetic Medication			Level of Adherence	
Informant	At baseline	12 month	18 month	12 month	18 month
1	Yes	No	No	Adherence	Adherence
2	Yes	No	(lost to follow-up)	Relapse	(lost to follow-up)
3	Yes	Yes	Yes	Relapse	Non-adherence
4	Yes	No	No	Adherence	Adherence
5	Yes	No	No	Adherence	Adherence
6	Yes	No	No	Adherence	Relapse

**Table 5:** Main themes and findings regarding informants’ motivation and barriers to adopting and maintaining lifestyle changes, based on interviews at 12 and 18 months.

12-Month Themes	18-Month Themes	Main Themes
Positive benefits from lifestyle change Ability to prioritise and plan	Benefits from lifestyle change Perceived threat of diabetes Goal setting	Achievement of results
Time and energy consumption Lack of flexibility Disruption of daily routines	Daily rhythms and routines Belief in ability to maintain	Coping with ongoing challenges
Commitment Social relations An opportunity	Commitment and social relations Daily rhythms and routines	Social support and relatedness
Freedom from medication Competence/diabetes self-management Support from healthcare professionals	Monitoring and support from healthcare professionals	Support from healthcare professionals
Change in attitude Ability to prioritise and plan	Determination Belief in ability to maintain	Identification with and acceptance of new lifestyle
